# Autism through cinema: co-creation and the unmaking of knowledge

**DOI:** 10.1080/09518398.2022.2025492

**Published:** 2022-02-07

**Authors:** Steven Eastwood, Bonnie Evans, Sebastian Gaigg, Janet Harbord, Damian Milton

**Affiliations:** a Film, Queen Mary University of London, London, UK; b Autism Research Centre, City University, London, UK; c Social Policy, Sociology and Social Research, University of Kent, Canterbury, UK

**Keywords:** Autism, neurodivergence, co-creation, archive film, proxemics

## Abstract

This article discusses the methodological approach of a collaborative research project situated at the intersection of autism and cinema. The Autism through Cinema project stages an encounter between the titular terms in order to challenge the neurotypical assumptions that underpin cinema as an apparatus, and to mobilise new cinematic potentialities. Structured over a period of four years, the project undertakes a series of collaborations between different disciplines (Film Archaeology, Film Practice, History, Psychology, and Sociology) and between autistic and non-autistic thinkers and makers, as well as in partnership with institutions external to academia. Methodologically the motivation for the project is to reverse engineer research methods used historically in film history, film making and psychiatry to produce inversions of perception, power and classification, and furthermore to explore what kind of cinema is possible if it is reconfigured through autistic experience.

## Introduction

This article discusses the methodological approach of a collaborative research project situated at the intersection of cinema and autism. The *Autism through Cinema* project stages an encounter between the titular terms in order to challenge the neurotypical assumptions that underpin cinema as an apparatus, and to mobilise new cinematic potentialities. Structured over a period of four years, the project undertakes a series of collaborations between different disciplines (Film Archaeology, Film Practice, History, Psychology, and Sociology) and between autistic and non-autistic thinkers and makers, as well as in partnership with institutions external to academia. A range of activities examines the capacity for autism to redraw the parameters of cinema and explore what kind of cinema is possible if it is reconfigured through autistic experience.

The relation of autism to cinema has been explored topically within the humanities in recent years as a means of raising awareness of contemporary and historic autistic lives and promoting self-advocacy. Phil Schwarz has suggested this about figures such as Glenn Gould and Alan Turing whose lives have been fictionalized in feature films (Schwarz, 2008). The representation of autism in film, like that in literature, is subject to scrutiny for recurring traits, motifs, figural flourishes and story arcs. The past two decades have seen a rise in the cinematic and televisual presence of autism, yet as Stuart Murray’s research identifies, post-*Rain Man*, cinema’s embrace of autism casts its image through a narrow codification or stereotype; broadly speaking, a savant of exceptional ability acts as a catalyst for change or redemption for a non-autistic character (Murray, 2008). Autistic characters, in Murray’s persuasive reading, operate as a narrative device providing a foil for the main protagonist, never knowable or relatable but performing a back-grounded difference. A sustained critical focus on representation, as Mark Osteen contends (Osteen, 2008) 02, has provided a necessary exposure and critique of the power structures that produce stereotypical iterations of autism. But its risks are that representational analysis of film evaluates autistic characters against their capacity to authentically represent autism and autistic people in the world, as though autistic characters must carry the burden of representing a way of being that can be cross-referred to an equally problematic taxonomy of gestures and behaviours. Moreover, to attend only to the representation of autism in cinema leaves unexamined the cinematic apparatus; that is, the ways in which a neurotypical cinematic language is produced through techniques of camera positioning, framing devices and editing techniques that naturalise performance styles and induct the spectator in a mode of interpretive inference. Faces filmed in close proximity, to take one example, are inscribed within the cinematic apparatus as privileged sites of meaning. In adopting an approach to cinema as system or dispositif, representation is but one facet of a perceptual training in neurotypical apprehension of moving image culture.

Methodologically the motivation for the project is to reverse engineer research methods used historically in film history, film making and psychiatry to produce inversions of perception, power and classification. The methodology is also an undertaking to work across various boundaries, involving autistic adults and young adults, carer groups and charities, cultural practitioners, and academics in the disciplines of visual culture, social sciences, and medical care. The aim, to be clear, is not to reduce disciplinary distinctions, but to borrow methodological tools that can be repurposed in a new context, a form of methodological prospecting that offers a commentary on the approach itself. In planning the project, the search for conceptual affinities and resonances across different fields was accompanied by a fascination with the stark differences in methodology; for example, the use of eye tracking in psychological research offered itself to a different set of questions and relationships in the context of film viewing and filmmaking. The realization of the project occurred to a large extent through the plan to import methods from one discipline into another, methods that are themselves changed by their relocation within a new paradigm. A second critical consideration for the project’s methodology shifts critical focus from acts of analytical interpretation of disabilities, often written from the outside, towards arenas of experimenting and doing, prominently in the co-creation of a community of practice (Milton, 2017b) defined by shared interest in autism as an alternative mode of communicating and experiencing the world.

To bring this point into focus, the methodology in the first instance imports an archaeological approach from the discipline of film studies into the domain of psychology. Psychology becomes through this approach a vividly visual discipline; archaeology reconstructs cinema and autism as they emerge from a shared milieu at the beginning of the twentieth century (taking Bleuler’s initial coining of the term as an origin of sorts), a period characterised by widespread investigation into the efficiency and communicative capacity of the human body. How bodies perform every-day functions and to what extent the individual’s agency in executing movements is evident to the observer, is a question for the nascent fields of cinema and psychiatry. In the early twentieth century, a silent cinema without spoken language strove to develop and market a gestural body language that communicated effectively. Contemporaneously psychiatry began to engage film as a new method of observation of patients’ idiosyncratic movement. Whilst autism in early medical film is not named as such, it is present in the capture of acts such as stimming and ticcing. Medical film and silent cinema share an enquiry into what can be understood of a range of bodily movements by, respectively, a scientifically mandated observer or a commercially mandated spectator. Whilst the disciplines of psychiatry and visual culture narrate the respective histories of autism and cinema as distinct, autism can be seen to emerge as the category of what cinema is not. Conversely, as Anson Rabinbach argues (Rabinbach, 1990), cinema becomes the domain where exemplars of human efficiency, self-knowledge and agency are modelled.

Autism gives definition to the language of cinema by operating as a foundational exclusion or negative presence. That autism has been storied through a series of contrasts leads M. Remi Yergeau to state that “autism has been essentialized and thereby made (un)known as a condition of opposing fields, as a condition that, in toto, defies.” Autism, they argue, is “that which contrasts with language, humanness, empathy, self-knowledge, understanding and rhetoricity,” notably preserving and defining the neurotypical as the model that autism mirrors in the negative (Yergeau, 2018, p. 2). The methodology in the first part of the research is defined by an archaeology attentive to the cast of the negative, the boundary that marks off what has been segregated and positioned as the backdrop against which the dominant neurotypical culture is animated. The use of archaeology is not only cartographic (in mapping correspondences across knowledge fields), but interventionist. When autism becomes a more prevalent term and diagnosis mid-century, medical films about autistic children emerging in the postwar period enter into the public domain. Film as a technology transitions autism from the enclosure of clinics into televisual and cinematic space as information films and topical documentaries with expository voice-overs. The task of the research is to bring a selection of these films for debate between autistic participants to collaboratively create a contemporary voice-over to re-interpret events, narrate what they see and comment on both the clinical practices and style of filming, or to respond in drawing or music to create an essayistic weighing of historical materials that have framed autism problematically.

As many have argued before us, the problematic nature of medical and psychiatric research that has sought (and seeks) “to know” through external observation and experimentation has produced a historically changing typology of the features of autism, a roster of symptom clusters that attempt to define and diagnose a “condition.” Each historical wave of research-reporting is presented as a form of disinterested knowledge available for translation into non-medical discourses and exercised creatively in fictionalized performances of the autistic subject on screen and in literature. Foundational to the methodology, perhaps above all, is the transformation of a research agenda through the work of neurodiverse authors and advocates of self-definition of autistic individuals and communities. The historical legacy of medicalized research and social care models positioned autism, until relatively recently, as a disability with a focus on identifying and describing the “deficits” and “impairments” associated with what is framed as “disorder.” Nosologically, autism has been defined as a pervasive neurodevelopmental disorder primarily characterized by difficulties in the domains of social interaction and communication, combined with sensory processing differences and a preference for routines in thought and behaviour. In recent years, however, there has been a shift away from this deficit model toward an understanding of autism in terms of differences in experiencing and engaging with the world. This shift has also involved a change in research practice, whereby autistic individuals increasingly cocreate understanding about themselves and everyone around them *with* research teams instead of serving as subjects of observation (Fletcher-Watson et al., 2019; Milton et al., 2019).

The programme of research in pragmatic terms unfolds broadly as four stages that each provide materials, inclusive of points of provocation, for the following stage. The first of these, as noted above, marks a period of research in entertainment film and medical film archives to trace a fascination with the communicative capacity of the human body at the beginning of the twentieth century, and again in the postwar era. Critical to the establishment of both neurology and cinema was the articulation of gestures as intelligible (the question of what could be understood from a body’s movement was key to silent cinema), or conversely so-called unintelligible gestures, such as stimming, were the object of clinical investigation. The second stage of research is characterized by a series of workshops operating as experimental hubs wherein practices of observation, modes of representation and eye-tracking techniques are deconstructed and reversed, whilst scenes of feature film sound and image are re-mixed and image scale and sequence recalibrated. The third phase of the project moves into collaborative film practice via the constitution of a filmmaking collective involving autistic and non-autistic collaborators. The methodology for the filmmaking shifts critical focus from acts of analytical interpretation of disabilities, often written from non-autistic scholars, towards arenas of experimenting and doing, adopting a co-creation model involving a community of practice, defined by the participation of autistic makers. The methodology underpinning the co-creation process will be explored towards the end of this article; however, a definition may be of use at this point. A 2018 report commissioned by Arts Council England titled *Cultural Democracy in Practice*, offers that: “Co-creation … is a term that reflects a mutually beneficial relationship, maximising the expertise of everyone in the room, to create a process or product that everyone has played an active role in.” Whilst the cinema, like many collaborative/industrial art forms, inevitably involves the creativity of many, the operation of bringing individual contributions together tends to be hierarchical, top-down, and, crucially, when it comes to autism, an act of “speaking for.” In the model put forward within the Autism through Cinema project, co-creation means playing to the individual strengths and aspirations of each collective member, and meaningful involvement in the process of filmmaking at all stages and levels, from concept development, through key production role apprenticeships, to collective contribution to the film’s authorship.

The research thus asks, what kinds of moving images are possible if they reflect a neurodivergent rather than a neurotypical experience of being in the world? Moving through the history of film in clinical and entertainment spheres, to experimental workshops and filmmaking, a central question is how autism can transform established cinematic norms. Across all aspects of the research, autism is regarded as the optic rather than the subject.

## Part one: film archaeology

An archaeological approach to the presence of autism in medical and commercial film, led by Janet Harbord, has a number of foundational features. Influenced by Foucault’s excavations into genealogy (Foucault, 1966), the idea of origins is supplanted by an archaeological examination of the conditions of possibility that enabled a statement, a technology or a diagnosis to become thinkable. In this project, the archaeological question enquires into how disparate technologies and divergent behaviours cohered and became sustained as, respectively, cinema and autism. Second, as media archaeologist Jussi Parikka (2012) contends, this approach is inevitably productive of minor or counter histories, observing trends and correspondences that have gone unnoted in the dominant accounts of culture and subjectivity. Third, arising from the first two features, archaeology evidences how a minor or repressed history gives shape to the dominant or major form, be that a definition of cinema, subjectivity or modes of cognition. Far from operating on parallel tracks, entertainment cinema was forged around the excluded absent-presence of medical film, whilst the concept of communicative normality was defined against the excluded term of autism.

Historians of psychology, psychiatry and neuroscience have long pointed out that all forms of human classification have a history, and that the meanings of all labels change over time. Diagnoses and categories are highly imbricated in the socio-political contexts in which they were developed, with autism as a prime instance of a category whose emergence and mutating meaning throughout the twentieth century is the result of historical buffering. The word “autism” was coined by the Swiss psychiatrist Eugene Bleuler in 1911 to denote one aspect of his definition of “schizophrenia,” a concept that he had also created (Bleuler, 1911: McNally, 2016). Building on the work of Bleuler, the Swiss developmental psychologist Jean Piaget wrote prolifically on autism in children in the 1920s (Piaget, 1928). Piaget regarded autism as an early stage of development that all children experienced. Bleuler and Piaget’s definitions of autism were similar to descriptions of a number of different styles of thinking in early childhood. The theory of autism was then primarily an important device for thinking through the early stages of general psychological development. The origins of the autism term are often wrongly ascribed to Leo Kanner in 1943, and whilst Kanner did describe autism as a specific syndrome, his understanding of the term was similar to that of Bleuler and Piaget, and his definition built on only a relatively small number of case studies (Evans, 2017).

An archaeological approach that attempts to trace the presence of autism in early medical film encounters a problem in terms of its referent. Autism was part of a proliferating number of classifications that arose on the one hand, from modernist methods of accumulating information of the population (using statistical and empirical practices) in the late nineteenth century, and on the other, the rise of the domains of neurology, psychology and psychiatry. As one of a number of diagnostic categories, it is not singled out for particular attention in medical film at this stage but appears as a likely presence in films of the first two decades of the twentieth century where the objective is to capture and parade physical idiosyncracies in motion. Tracing the use of film in European medical archives it is possible to reconstruct a network of influences that ripple out as a series of concentric circles from Jean-Martin Charcot’s work at the Salpêtrière Hospital, Paris. As Didi-Huberman has shown in what is now a classic study of hysteria, the disciplines of psychiatry and photography shared an intimate reciprocal relationship. The same may be argued for the relationship of film and psychology in the work of Albert Londe, Vincenzo Neri, Camille Negro, Gheorghe Marinescu and Jean Comandon. Where hysteria presented as theatrical display, the cluster of so-called disorders that manifest in idiosyncratic movements such as stimming and ticcing were documented as aberrant details of a pathology that may otherwise go undetected. The use of film in early clinical filmed contexts attempts to capture the singular instant of a symptom’s presentation. Marinescu, for example, was less invested in the projected film as an evidential material and more concerned with the film’s negative as a document that he studied and marked up.

It is important to engage with the wealth of film material produced by neurologists and psychologists from the late 1890s in order to understand its influence on new theoretical approaches to the workings of the mind from this period. Film technologies served an important role within the field of developmental psychology and psychiatry that an archaeological approach excavates with an eye attentive to the topic of autism. Indeed, what is clear from this early period is the mutual constitution and legitimization of film as a serious practice of documentation, and the knowledge claims of nascent areas of medical expertise, such as psychiatry and neurology. When autism came to be defined and shaped as a unique syndrome, film obtained through this process greater prominence as an instrument of objective documentation. The importing of film into medical practice in the early twentieth century stimulated new forms for scientific modelling in psychology, enabling in the decades that followed an increasingly visual model of normative psychological states as offered in the work of Èdouard Claparède, Jean Piaget and Arnold Gesell. By providing visual evidence of the “typical” and “atypical” movements of developing children, these films inspired new ways to study and reflect on the unconscious and psychological causes of different bodily movement, or what later came to be described as “behaviour” within later models of behavioural psychology. In 1934, Gesell claimed the film camera was the perfect scientific instrument to prove his theories on psychological development. In his opinion, recorded infant movements could be used to validate all theories of psychological motivation and to demonstrate differences across population groups. He believed that cinema had the capacity to get deep inside the human subject in a way that no other scientific method could.’ As Scott Curtis has argued, the use of film as an instrument of evidential knowledge was fundamental to Gesell’s approach to developmental psychology, which relied on observational practice in order to elicit internal subjective states (Curtis, 2011). That this method enacts a shift from observation of activity to a deduction of the subject’s thought processes, exemplifies the way in which film’s formal qualities of documenting events could be harnessed to medical agendas.

In the postwar period, from late 1950s, there was a major shift in approaches to autism and film concurrent with wider changes in the organization of institutional care and education for autistic children and adults. New ways of representing and articulating autism and autistic differences became more dominant in film where observation of patient gestures was given lengthier and more individuated film treatment. Patients were filmed interacting with medical staff in contexts of care where an environment of play materials and toys offered itself to experimental scenarios in front of the camera. As Bonnie Evans has argued, one of the most important films to have influenced current definitions of autism is *Aspects of Childhood Psychosis*, made by the child psychiatrist Elwyn James Anthony and produced at the Maudsley Hospital, London in 1957. The film was shown at the International Congress in Psychiatry in Zurich. Four years earlier, Anthony and his colleague Kenneth Cameron had established a clinic for children at the Maudsley Hospital with the aim of further understanding precursors to adult psychosis. Their theoretical approach to autism drew strongly from the Bleuler and Piagetian tradition, as opposed to Kanner’s approach. However, the main objective in making the film was to classify various behavioural presentations that were associated with children who did not adopt typical forms of social engagement. The film was based on seventy children who had attended the Maudsley Hospital and who Anthony regarded as complex cases that required in-depth psychological understanding. It is significant that this film was shown at an international event in Zurich in 1957, (Anthony had studied with Piaget at the Institut Jean-Jacques Rousseau where Claparède had pioneered film production in the 1920s, and so had many international links). Just a few years previously, the World Health Organisation had published a report calling for the closure of long-stay institutional care homes and for the improvement of community mental health services (WHO, 1953). It was this report that encouraged a growing number of Western countries to close down asylums and similar institutions. This was a major social and political transition that encouraged new approaches in many psychological and neurological sciences. In particular, it encouraged professionals to study, observe and film children and adults who were going to be entering community settings once new laws were enacted concerning long term institutional care.

There is a further point about the significance of Anthony’s film at this historical moment. As a visual record used to rehearse and interpret the key symptoms of autism (located within the broader umbrella of childhood psychosis), the film provided a diagnostic toolkit transferrable to other national contexts. This in turn paved the way for a consensual definition of the term autism, an international agreement within the psychological community of behavioural indicators. The film opens with an explication of its classificatory aims in a series of intertitle cards. The first half of the film shows brief excerpts of children in a large playroom, their activity cut down to ten or twenty second segments. These vignettes of actions and interactions with the object world (and occasionally medical staff) are each associated with a different aspect of what Anthony termed psychotic “continuums.” This was a precursor to Lorna Wing’s autistic “spectrum,” and Wing was to reference Anthony’s work in her creation of the “triad of impairments.” Anthony’s concern to capture and label a range is evident in the multiple illustrations of behavioural types, each example trimmed back to the symptomatic moment. Behaviours such as headbanging, hand flapping, or repetitive movements such as sifting sand through fingers, or repeatedly touching lights and bright objects, are offered interpretations and labels. One sequence depicts, in the language of the intertitle card, the “turbulent upheaval and wild tantrums and extreme fearfulness” of a girl rapidly moving her arms, grabbing at objects and trying to bite her own foot. The child’s behaviour does not necessarily correlate with the description, but rather is framed and parsed by the larger method of extracting “key instances” from a sequence of activity, a privileged moment that is clinically invested only retrospectively.

Clinical films, along with many other utility films, have an occasion, a purpose and an addressee, and as Hediger and Vonderau (2009) argue, their meaning derives from, and is useful for understanding, the institutional power and practice of which they are part. *Aspects of Childhood Psychosis* is a striking example of how children’s actions and movements were filmed as part of a larger project of psychiatric epidemiology, with autism emerging in the postwar period as a dominant descriptive category (Evans, 2017: 2018). In addition to reading this film in the institutional discursive formation of its time, an archaeological approach brings into view that which has travelled in time but may not have attained visibility in the moment of its production. At certain intervals in the film there is a burst of activity where a child appears to exceed or even to escape the framework through which she is observed. In one sequence, the camera follows a girl in the grounds of the hospital as she dances amongst the undergrowth and rocky terrain. She appears to be exploring the relationship between the shapes that her body can make and the various features of the landscape. An activity that is immersive, exploratory and sensorily experimental, the sequence is reminiscent of films made in the previous decade by the avant garde artist, Maya Deren, such as *A Study in Choreography for Camera* (1945). A body, in Deren’s film, is released from the confinement of theatrical space, moving instead through landscapes of a forest, a domestic interior and a piazza to expose the relative scale and structural properties of the human form against the material environment. The child in Anthony’s film similarly extemporises an environmental relationship as she rocks, sways and hops. Approaching medical film as a transmission across time, it reveals a register of gestures that might be called, following Julia Miele Rodas’ exposition of autistic language, an autistic poetics of the body. The comparison is instructive: Miele Rodas returns to an historic clinical text, Kanner’s “Autistic Disturbances of Affective Contact” (1943), and re-reads the examples of autistic language use as rhetorical devices. These she groups and names as five categories of rhetorical autististic poetics comprising ricochet, apostrophe, ejaculation, discretion and invention. The reveal of Rodas’s playful intervention across disciplinary lines is this: that the rhetorical qualities to be judged historically and currently as indicators of a cognitive and communicative deficit in a medical context are the same as those that are prized in another, here the context of revered literature (Rodas, 2018). Clinical film of autistic children’s gestures provides a similar opening to archaeological revision.

In summary, if the first stage of the project is characterized methodologically as the identification of autism in film archives and the exploration of film’s role in shaping the storying of autism medically, the reverse engineering of this approach takes place in a number of workshops with young autistic people who are invited to engage with and respond to the historical films. A first workshop took place in 2020 at Project Artworks, an artist-led organization working with young people with complex needs and their caregivers, inviting autistic people to view an exhibition of film extracts. The workshop took place over three days with visitors offered an opportunity to discuss the films, speak directly to camera, to write or draw a response, or to simply wander through the rooms. Out of the discussions that took place, counter narratives of the medical films began to emerge, speculating from the point of view of the children in the films. The responses in many ways confirmed Damian Milton’s argument of the double empathy problem (Milton, 2012): autistic people understand each other better than neurotypical people understand them, and vice versa, suggesting that empathy exists within groups rather than between them. The questions raised by this preliminary research has been continued online, inevitably given the current limitations on in-person encounters, with the further ambition of producing a response as a repurposed film. The shape has yet to be determined, but possibilities include an essay film where participants select sequences of the films, effectively remixing the original materials and bringing new connections to light. The creation of a sound track raises questions of language: sequences may be re-voiced or written as subtitles, with other creative options extending to mixing documentary images with soundtracks from fiction films. The method of re-purposing these relatively obscure films, that have nonetheless been significant in the history of autism as a diagnostic category, illuminates somewhat starkly the pathologizing of autistic idiosyncrasy, a practice critiqued and discredited in the realm of advocacy and increasingly in the academic domain.

## Part two: challenges to the “outsider” narrative of autism: nothing about us without us

Perhaps reflecting philosopher Ian Hacking’s (2009) notion of “bio-looping,” the first published personal accounts of autistic people began in the 1980s with Temple Grandin (1986), a potential made more possible with the broadening of what it could mean to be autistic to that of a wider “spectrum” (Wing & Gould, 1979). In the 1990s people who had been diagnosed as autistic started to attend autism related events and conferences and build their own online networks. This built the foundation for the autistic self-advocacy, autistic rights, and neurodiversity movements (Milton, 2020). From the seminal writings of autistic activist Jim Sinclair (1993/2012, 1999/2013) and a focus on insider accounts and participation, autistic community and culture has often been set against dominant medicalised pathological models of how to view autism itself. “… right from the start, from the time someone came up with the word “autism,” the condition has been judged from the outside, by its appearances, and not from the inside according to how it is experienced.” (Williams, 1996: 14). This challenge to established ways of defining not only autism, but how to help and support autistic people has been described as the “neurodiversity paradigm” often contrasted with what is framed as a “pathology paradigm” (Walker, 2014). Where variations in neurology, development, behaviour, and ways of being in the world are not purely seen in terms of individual impairment and remediation, but socially situated with an emphasis of social accommodations and social change (Arnold, 2012). It is clear to see the influence of both social model approaches, as well as rights-based approaches to disability activism that have laid the foundations for much of the concepts that have flourished within these accounts.

Central to these critiques have been the need to listen and involve autistic people in the decisions affecting their own lives (Milton & Bracher, 2013), to destabilise the idealising of normalcy regarding what constitutes a good quality of life (Milton, 2017a), the use of unwanted remediation techniques and interventions (Milton, 2014a), as well as discussions relating to dominant theoretical accounts such as theory of mind (Gernsbacher & Yergeau, 2019; Milton, 2012, 2014b; Yergeau, 2013). Alongside the work of autistic activists and scholars, were scholars working in the field of critical disability studies, often having a personal connection to an autistic person in their lives through family or work. The term “Critical Autism Studies” was coined in 2010 at a seminar day held in Canada, and was followed by another in Sheffield, UK in January of 2011. This burgeoning field includes autistic scholars who subscribe more to a neurodiversity model, as well as those with radical ideas against the notion of autism being used as a diagnostic category (Timimi et al., 2011).

Much of the criticism of dominant models is not anti-science either, indeed there are autistic scientists who have been highly critical of the ethical and epistemological standards within autism research (see e.g. the work of Dawson & Cowen, 2019). Recent studies have shown that the concerns of researchers and research funders do not always match well with community concerns (Pellicano et al., 2013; Fletcher-Watson et al., 2019). In the UK, the Participatory Autism Research Collective (PARC) was set up in 2015 to address some of these issues and to raise the profile of participatory research in the field (Milton et al., 2019). PARC aims to build a community network where those who wish to see more significant involvement of autistic people in autism research can share knowledge and expertise. PARC follows in the footsteps of previous autisticled projects, such as the *Autonomy Journal* and the Theorising Autism Project, who have been campaigning for more participatory autism research. In 2019, the PARC network collaborated with the Autism Ethics Network and researchers based at the University of Kent to hold a conference combining concerns around participation and to explore research in the field of autism across disciplines, including work based in the Arts and Humanities, particularly featuring the “Playing A/Part” project (2020).

Recently there has been something of a convergence of differing disciplines around issues with concepts derived from autistic scholars such as monotropism (interest models) or the double empathy problem (Milton, 2017) and enactivist or predictive-coding models applied to autism (Bolis et al., 2017; De Jaeger, 2020; Jurgens, 2020). As Ian Hacking once pointed out of pervasive narratives surrounding autism: “They are creating the language in which to describe the experience of autism, and hence helping to help forge the concepts in which to think autism.” (Hacking, 2009, p. 1467). Hacking suggests that biographical accounts of autistic experience, such as Grandin’s *Emergence*, are not merely demystifying texts giving access to autism from the “inside,” but rather openings onto new possibilities for experience for which we have no adequate linguistic structures, and which should not and cannot be reducible to autism as an adjunct to human experience: they are for all of humankind. New language is required, and this does not only apply to spoken language, but also to the assumed naturalised language of cinema as the language by which autism must be depicted and described. The cinema language we have has restrictive norms of body gesture, body language, social interaction, configurations around the face, and so on. Autism cannot be encountered with these existing film forms. A progressive film language is needed, one that resists, exposes and redefines the naturalized language of cinema. Such a cinematic form would not look at autism, but look with and through autism.

Autistic people represented in film have often been displayed as an exotic other, or a plot device, further embedding ableist assumptions and stereotypes. Autism is typically represented within a deficit model, as a deviation from an idealised normalcy, with filmmaking opportunities falling back into a pre-existing cinema language and production model that by design excludes autistic artists and makers. Whilst the medicalisation of autistic ways of being is often seen in need of remediation and intervention, in this project it is the cinema that is identified as requiring remediation and intervention, from an autistic perspective. This project has sought to interrogate and innovate film practice, with various activities including workshops, online surveys, podcasts and co-created filmmaking having numerous potential pedagogical applications. Much like recent moves to decolonise the curriculum, one of the project aims is to de-neuro typify the film studies curriculum. The critique of canonical autism-subject films within the podcasts, surveys and workshops, and attention given to lesser-known titles in the touring film season Autism and Cinema, suggest specific film titles for inclusion within Film Studies/Media Studies/Disability Studies modules and programmes, with the aim of interrogating ableism in education settings. The Representations of Autism workshop survey outlined below has a specific use as an interactive tool for challenging the savant/medical model view of autism – the workshops use neuro-divergent perspectives as a tool for deconstructing normative cinema structures. The project also involves the inclusion of disabled people in film creation and critique (the Neurocultures Collective, the Autism through Cinema podcast), proposing new cinematic forms built around progressive ideas of the human subject.

The pedagogic application of the research could have wider reach beyond the academy, bringing the film industry and art world (producers, programmers, funders) into dialogue with an autistic constituency, informing practices of inclusion, and channelling an autistic influence into cultural production and exhibition. The project reaches out to autistic communities interested in the interrogation of the history of autism and diagnosis, and in how neurodiverse co-creation, through mutual learning opportunities, can reframe perceptions of an autistic sensibility. The research also seeks to reach general arts and film audiences who are often exposed to limited narratives of autism and may benefit from a more nuanced understanding of neurodivergence.

Film representations of autistic people, or even fictional depictions used for training purposes in relation to those working with autistic people, often frame autism within a purely medical model narrative. Through our engagement with clinicians, psychologists and medical constituencies the research findings stage a debate surrounding the notion that diagnostic testing is an end in itself.

## Part three: neurodiverse perspectives on cinema

Between 2019 and 2020 a number of film workshops were held to develop the findings of the film archeology as they might be applied to commercial fiction film. Each workshop was designed to act as a building block towards the formation of a community of people whose ideas, experiences and skills will come together in the co-creation of new film practice.

The Screen Dynamics workshop held at Queen Mary University December 2019 and led by Dr Sebastian Gaigg (City, University of London) and Steven Eastwood (QMUL) invited members of the autistic community to interact with a set of video stations, each addressing a key aspect of cinematic language. Rather than autism being placed in the laboratory, the format of the workshop puts the orthodoxies of the cinema under the microscope, as seen through autistic perspectives. Visitors could wander between five stations, each of which created a context for critiquing the cinema’s representational norms. A number of dynamics and discourses pertaining to autism and to how the cinema renders the human body, the face, and the social norms of the film scene, became discernible.

The Proxemics Station considered cinema sequences involving faces, bodies and crowds, and how human subjects are commonly positioned in relation to each other on the screen, and in terms of closeness to the camera and therefore dominance within the film frame. This included the wide vistas and dramatic extreme facial close-ups of *Once Upon a Time in the West* (Sergio Leone, 1968) and the highly intimate closely photographed sequences of *Faces* (John Cassavetes, 1968), amongst others. This station also staged how close we comfortably position ourselves in relation to the actual cinema screen and its enlarged scales of faces and vistas, by tasking visitors to stand where they felt most comfortable in relation to the screen. This station drew attention to how entertainment cinema across genres and eras can readily be typified by the close-up of the readable emotions of the emoting human face.^1^ Indeed, the assumed social conventions of the face-to -face encounter is upheld within continental philosophy, for example in Emmanuel Levinas’ work, where the face is that which appears in our field of vision as a question that demands a response and answer (Levinas, 1985). Opening up Levinas’ concept, Gilles Deleuze considers the face as the site of affect or an opening, a black hole of subjectivity, a consciousness that can never be accessed, goes so far as to name the cinematic close-up of the face as the affection-image (Deleuze, 1986). Deleuze explores the face as predicated on constructs of personality and subjectivity based in social consensual and habitual norms of empathy, reaction and interaction.

However, a considerable body of research has accumulated since the 1990s which suggests that the face does not hold the same prominence and salience for autistic individuals as it does for neurotypical audiences. Autistic observers tend to spend less time looking at the faces of characters in film sequences, and during direct interactions with others often prefer not to make eye-contact. Under the “deficit” discourse that has dominated the research literature for too long, these observations have led to endless debates about the underlying causes of this difference in observing the world: is it the result of a broken face-processing module in the brain, or a lack of social motivation, or an impairment in so-called “Theory of Mind” that hinders autistic individuals from accessing the thoughts and feelings of others through the face? These debates have entrenched thinking to such an extent that more important questions have rarely been asked. What *do* autistic individuals prefer to look at, and *why*? What meaning do they derive from scenes and film sequences that neurotypical observers may not be privy to or overlook? There are important exceptions in this literature that have considered questions beyond the ‘deficit’ framework. In particular, there is considerable work on ‘enhanced perceptual functioning’ in autism, which essentially shows that autistic observers have an increased capacity to process sensory information. This generally means that an autistic person takes in more information but also that this can cause difficulties in filtering incoming information effectively. The Proxemics Station provided opportunities to explore these questions by considering other “screen bodies” not predicated on the face as question or emotion, a cinema instead constructed around or adjacent to or behind the face, being facially *besides* or *with* or even *without*, rather than frontal and towards the face.

The Event Boundaries station invited participants to work with a video editor to examine a series of film scenes and identify when a perceived discrete event has taken place. The very construct of a scene in a film narrative carries with it economies of description and expectation. For example, a screenwriter will commonly assign an umbrella meaning to a scene. A film scene establishes place, begins where it needs to and ends when it needs to, without surplus and unmotivated screen time and activity. Scenes are densely populated with micro-events, and these might include physical gestures, call and response actions, peripheral details such as the movements of extras in the background, passing cars, doors closing, the movement of objects, as well as of course the intentional activities of focal subjects such as actors and dramatic actions. The functions of the Event Boundaries station was to bring into focus our many attention differences, and how normative cinema operations are predicated on inferential reading, body language, and folk psychology (whereby the implied meaning of a sequence is to be deduced from a series of external signs and micro events). These short hands to meaning can be exclusory, particularly to neurodiverse viewers who may find such social and deductive codes confounding. Asking individuals to mark where they perceive film events to have taken place highlights assumptions made by cinema makers about how audiences parse unfolding events. The station thus draws attention to conventions within film practice, and enables us to consider the ways in which the very form of the edit – between scenes and within scenes – as well as the gestural shorthand of intentional bodies on screen, may for specific audiences undermine film narrative clarity. The station also introduces new concepts for film form as recognised and/or required by autistic audiences, and how these forms might disrupt existing norms. These include how an edit may be in service to images which repeat and “stim,” or mark an event-boundary not predicated on character or plot. Similarly, the Reframing Station involved participants working with a video editor, choosing to enlarge or loop and repeat an area of the screen within specific film scenes, so as to privilege background events or peripheral details taking place beyond focal human subjects. The re-framed and re-edited sequence make visible a fuller and more populated frame, magnifying the cinema to reveal hidden and unintentional elements brought into focus through an interest in objects and patterns which exist behind or beside the focal areas of a film’s narrative.^2^

The Eye-Tracking station provided further opportunities to question conventions in film practice by providing an opportunity for conversations about points of interest across a range of different onscreen spatial arrangements residing in different techniques spanning action sequences, comedy, non-Hollywood cinema and more. These included the kinetic camera attitudes in *Mad Max: Fury Road* (Miller, 2015), classic foreground/background deep focus arrangements in *Citizen Kane* (Welles. 1941), rotating camera movements in *The Boat* (1921), an early Buster Keaton short, and the arguably less directed gaze produced by the long takes and wide shots in *Playtime* (1967) by Jacques Tati. In psychological research settings, eye-tracking is often used as a tool, primarily to observe and quantify how observers direct their attention at various details of static or moving images. By contrast, the current workshop utilised the technology to generate visual representations of such patterns of attention that observers of film sequences could watch back and explore. This reverse-engineering of the eye-tracking methodology provided opportunities for rich conversations with audiences about points of interest in film sequences and about shared versus more personal viewer experiences. For example, the technology might reveal points of attraction that do not necessarily privilege the human face, but rather backgrounded objects or visual details that the filmmaker did not intend as significant to the unfolding narrative. Conversations could also turn to the relative “effort” that different film techniques might demand of an audience (e.g. effort in following points of interest and or in “knowing” where one should direct attention). The station thus also provided an opportunity to put film practice under the lense by revealing differences in audience experience across different film extracts that might vary on dimensions such as the number of scene elements that could become the focus of attention, or the speed with which shifts in attention are required to keep track of movement or of information across cut scenes (or to discover new points of interest). The station thus afforded opportunities to, almost literally, experience film *with* the audience and *through* their eyes as a means of exploring film practice. Moreover, the exercise enables us to consider formal and cultural differences in cinema styles, for example, how one kind of cinema is defined by the emoting face, while other cinema iterations present the human figure far away from the lens (Tati), so that the face need not or cannot be read.

The final station tasked participants with re-mixing audio tracks on a number of movie scenes including *Captain Marvel* (Anna Boden, Ryan Fleck, 2019). Working with a sound engineer, visitors to this station could use a mixing desk to mute and re-arrange dialogue, music score, environmental noise and discrete sound effects, in doing so producing new audio hierarchies. This interactive zone enters into dialogue with the concept of autism friendly cinema screenings, but rather than adopt the somewhat blunt instrument of raising the lights and reducing the overall volume of a film (acknowledging that both measures can significantly reduce the anxiety some autistic people experience in the movie-going environment), this station enabled participants to learn about how layers of film sound produce aural focal points and intensities, and to intervene in this operation and re-engineer the sound based on personal comfort and curiosity. This opens-up possibilities of a cinema less enshrined in spoken dialogue exchanges and the insistence of effects, one more nuanced and based on sonic subtleties, environments and presences.

The focus on proxemics and gesture, or what is ordinarily referred to as body language, fore-grounds an historically changing code operating at an unconscious level, which is institutionalized as a normative set of rules in film production and film viewing. The cinematic rules governing how bodies are positioned for filming and put into relation with one another in editing is a specific visual language that actors and audiences had to learn and continue to observe, characterised by eye contact continuities across shot and reverse shot montage, and by event boundaries such as scenes, coded as having the specific function to advance character and plot: dynamics that may, or may not, hold the same meaning for autistic people, who have said they find this implicit film language difficult or even nonsensical. To shed light on the assumed norms of social and cultural distance, proximity, and comfort, the experiments with proxemics in a constructed setting allowed viewers to enter a room without seating to watch a number of film extracts with differently scaled sequences from wherever they felt comfortable. Desired viewing distances provide useful insight into whether differently scaled sequences (very close-up faces, middle distance figures, long lens crowds, and different styles of editing) are experienced as too intrusive or invasive, or led audiences to want to retreat from the scene, or invited and thus drew audiences closer. Each of these stations serves not only to give analysis to the assumed norms of narrative cinema (e.g. norms of the audio prominence of dialogue within dramatic scenes, or standards of shot structures predicated on human physiognomy, e.g. a close-up shot is measured by the face) but also to reveal potential within the film frame, and agency for the cinema to shift its normative language. The cinema, arguably, is a social construction, and its aesthetic and functional habits can be deconstructed and reconstructed.^3^

A further workshop, the Representations of Autism survey run by Dr Damian Milton (PARC; Kent) and Steven Eastwood (QMUL), was made open to an invited group of autistic individuals and carried out online in July 2020. Designed to be inclusive and accessible, participants were asked to consider the forms of description documentary and fiction has given to autism. The survey featured extracts from twelve examples, including Hollywood narrative, factual television, animation, and work by autistic filmmakers.^4^ Contributors were asked to describe the things they found accurate and inaccurate in the samples and to consider popular misconceptions surrounding autism, and how they may be challenged. At the end of the survey participants were asked what they would do differently if they were the filmmaker. Initial findings culled from survey entries speak to how fiction films about autism tend to resort to lazy and inaccurate stereotypes, and documentaries can focus on negatives or be over simplistic.^[5]^ Both tend to try to describe what it’s like to be autistic and this can be reductive. Respondents articulated the need for film-makers to avoid ableism and tropes of victim culture, and steer clear of simulating disability and autistic experience (something autistic activists have fought against).

## Part four: the neurocultures collective: a co-creation filmmaking model

The workshops held to date have enabled the project to engage with a number of autistic individuals and to collect a range of formal, aesthetic and conceptual tools to take into the co-creation phase of the research.^5^ In order to embark on co-creation, a film practice methodology is required and a mechanism for collaboration, what we are calling *The Neurocultures Collective*.^6^ This method of co-creation deploys an action research participatory model, using film practice as a research tool to integrate the views and sensibilities of autistic people, and to create an alternative visual language from the perspective of autism. Based on findings and dialogues to date, the practice starts from the understanding that documentaries need not merely be about character studies and talking heads, they can focus on environments and communities (e.g. a “neurocultures” community) and can feature an ensemble of characters. Although rarely articulated in mainstream cinema, moving images can involve interests beyond and behind the human face, as well as sensory descriptions, affordance to objects, use of gentle repetition, and stimming adopted as an editing principle (hence the term Stim Cinema). These are forms which have been taken up by the avant-garde, by artist-filmmakers, and, significantly, by autistic filmmakers.

In order to create the film practice, project lead Steven Eastwood will work with an advisory panel to design the participatory co-creation process and then bring together individuals who have participated in the workshops. This will lead to the formation of the collective, who will develop the film’s content. A number of the collective will take up apprenticeships on the film production, others will perform in film, and several participants may emerge to co-direct the film(s). Such a model of participatory co-creation is being increasingly taken up by art projects involving marginalised communities, as well as by those who wish to embed principles and methods of equity and inclusion in the process of making. Katarina Cizek and William Urriccio (The MIT Co-Creation Studio) recent published *Collective Wisdom*, an exhaustive study which draws upon extensive field research and interviews with organisations adopting participatory practices.^7^ These include, in the US, the Detroit Narrative Agency (DNA), whose fellowship program supports Black and Brown filmmakers in Detroit to develop short films and generate community impact strategies. The study builds upon the work of scholar Patricia Zimmermann who also adopts the term “reverse engineering,” as a means by which to create new vantage points onto the complex ecologies of documentary practices, and moreover, as a way to imagine ways how the very concept of documentary might evolve and be restructured to include and mobilise communities.

Co-creation is emerging as the characteristic practice for an era when disenfranchised and marginalised groups have access to the technological resources to finally be able to draw upon networks of distributed agency. Cizek and Uriccio’s study finds in collective practices a common desire to question truth manufacturers, challenge power and form more nuanced descriptions of the world, although the authors point out that co-creativity remains under-reported, can be vulnerable to abuses, and can too easily be siloed as “socially engaged” or “community arts” practice (Cizek and Uricchio, 2020). For example, established artists and documentary filmmakers can enter into vulnerable constituencies in the name of co-creativity without in fact relinquishing authorship or considering the impact and legacy of often short-lived engagement and intervention. Conversely, individual creativity can become flattened by horizontal democratic production systems. The contemporary art world arguably has a systemic problem with difference, all too often side-lining art made with and by marginal groups into the ghetto of education programmes or access and awareness campaigns. This is not to suggest that these initiatives are not without validity, but they rarely permit collectives, or individuals such as autistic makers to exhibit as equals.

The Collective Wisdom study contextualises this cultural apartheid as the product of centuries of top down, single author, meaning–making art, whose articulations represent the power of a single authority. What unites initiatives like DNA and UK organisations such as Project ArtWorks (PAW) is the objective to resist “extractive, harmful, commodifying practices” (Cizek and Urriccio) and enable those disinclined to perform their trauma to instead benefit from deep-listening and inclusive systems, working with just models of decision. Project Art Works for example, supports and facilitates the independent art practices of a number of artists who use their studio spaces and Parachute Club, including Sharif Persaud, who had a recent solo show of paintings and film artworks at Autograph, a gallery in East London. PAW’s 2019 *Explorers: Illuminating the Wilderness* is a programme of art devised to challenge perceptions through creating supportive and deeply affecting interactions between neurotypical and non-neurotypical artists and individuals, collaborating across a range of art media including a tactile, shared and exploratory form of filmmaking conducted in wild landscapes.

These progressive and inclusive mechanisms for filmmaking and other arts practices raise a number of logistical, ethical and legal questions, particularly in terms of authorship and ownership. The Neurocultures Collective is about challenging existing social structures and disrupting restrictive film production structures. There are some existing historic models for working in this way, from the “cottage industry” of underground film communities to the collaborative activist endeavours of the proponents of Third Cinema, who created collectives in order to reject the “genius author” model and democratise the filmmaking process. It is important that the mode of filmmaking be both open and reflective, allowing for subjects and unfolding events to determine what is on screen, by way of continually filming, reviewing and refining form and content. However, boundaries and structure can be very important for people who identify as autistic. Some autistic people have articulated a need for structures in order to break away from them. Unanimous decision making is difficult in any setting. For this reason, the project intends to work with specific decision-making systems, for instance using hand gestures as well as verbal language, and adopting the “assent, consent, dissent” system developed by Project Artworks. The co-creation model that has been developed following the workshops and in consultation with partners and advisors exists as a starting point, but it will be refined through discussion with participants.

The Neurocultures Collective will co-create a feature length film, working title Neurocultures, and a multiscreen video artwork, with the working title Stim Cinema. Some collective participants may appear in the film as performers, others emerge to co-direct, or engage in specific areas of production and post-production by taking up apprenticeships. Some participants will have existing conceptual frameworks for the cinema upon which to draw, whilst others may contribute to the process of co-devising with nonverbal modes of expression and communication, for example using the camera as a means of seeing and listening, gesturing and drawing. It is possible that the creative collective process may determine that autism is not the subject of the film at all, but instead the method the film adopts for seeing. Members of the collective are thus able to turn the tables on the historic misrepresentations of autism, and lever open some of the orthodoxies and assumed neurotypicalities of the cinema. The film practice therefore attempts to rethink the cinematic social contract, working with autistic perspectives as the optic, moving away from symbolic or inferred meaning and towards the experience of embodiment, the joining together of things into networks, and an apprehension of worlds as sensations, patterns and rhythms.

## Conclusion

One of the notable features of the project is that its methods are appropriated from film, psychology, history, archaeology and collaborative activism, which in turn highlights the anomaly that the moving image as it relates to embodied states has obtained less prominence in debates about autism than verbal language. In the contemporary landscape, autistic rhetoricity has been the subject of reclamation and a thoroughly revised understanding of speech and writing patterns as flows and ricochets found prominently in the work of Yergeau and Rodas (among others). Such language-oriented work directly counters the diagnostic mis-apprehension of autistic rhetoricity located in diagnostic classifications and circulating more broadly in educational settings. This research project approaches a similar horizon in the field of vision and embodiment, reaching for what Dinah Murray has named “an ecological, embodied, enactive and exploratory account of minds,” an account that takes the movement of the body as its touchstone. A significant part of our methodological has approach has been to appropriate and repurpose the visual tools that have been used to formulate deficit accounts of an autistic modality: observational medical film, the pedagogic documentary film and eye-tracking devices are each freighted with responsibility towards the historical pathologisation of autism. Yet in returning to these methods of knowledge production, it is possible to make visible not only the vested interests of such research but to bring to light the potential of autism and cinema, of cinemautism, that was foreclosed.

Autism through Cinema initiates a re-telling of the story of cinema with and through autism that runs like this: for the twentieth century the moving image became the preeminent form for describing human behaviour. As the cinema rose to become a mass media, modern psychiatry and concepts of psychology also emerged and became popularised. The communicative norms of the human body as speculated, observed, measured and established by the burgeoning paradigm of psychology in the early part of the century ran parallel to the forms the early cinema took in describing human subjects and their encounters with others. Exemplars of the early expressive form, such as Chaplin and Keaton, whose bodies did not conform and whose angular and often berserk movements markedly separated them from others, could not translate their physical language into the era of the talkies. Where pre-sound cinema relied upon overtly gestural bodies, the advent of sound in movies pushed meaning into the words speaking subjects said to one another. The apparatus for sound recording and increased lighting and camera structures in the 1920s led to the necessity of blocking performer comportment and subsequent fixities of body movements and gestures. Structures such as the over-the-shoulder shot and the eyeline match helped tether subjects to one another and set out spatial and relational habits for the viewer. The close-up of the face became the anchor of the cinematic image. As the cinema entered its golden era in the 1930s, the proxemities and behavioural conduct it disseminated came to stand for our communicative capacity. Simply put, uneconomic gestures, hesitancies in speech, and, explicitly, disability, had no space in the film frame. As the research continues, the endeavor remains, not to produce a cinema of disability, a parallel track or an alternative silo, but to expose the neurotypical foundations of the cinema that exists and to bring into being a more complex, diverse and imaginative cinema of the future.

## Data Availability

For the purpose of open access, the author has applied a CC BY public copyright licence to any Author Accepted Manuscript version arising from this submission.

## Notes

1 A 2019 promotional trailer for Curzon Cinema’s Curzon-on-Demand comprises a montage entirely consisting of the emoting faces of film stars/film characters from titles within the online service.

2 Recent works of artists’ moving image privileging backgrounds, trees, plant life, wildlife, and extras include, Lucy Clout’s The Extra’s Ever Moving Lips, 2014 and Eija-Lisa Ahtila’s Eija-Liisa Ahtila, Studies on the Ecology of Drama, 2014.

3 It is worth noting that social construction and symbolic interaction theory, and the work of Erving Goffman, has not been taken up within critical autism studies.

4 The architecture of the survey is indebted to the work of Stuart Murray, whose Representing Autism: Culture, Narrative, Fascination (2008) gives thorough attention to the all too often unhelpful and inaccurate autistic types and tropes within popular culture film and television.

5 Subsequent workshop activities have included creative home kits, boxes sent out during the 2020 lockdown period to a small group of people. These involved a series of prompts to produce artworks (drawings, comic books, storyboards, sound recordings, and more) drawing on the everyday life and environments of participants. The findings and outputs of the Screen Dynamics workshop, Representations of Autism survey and the Creative Home Kits will form the basis of subsequent publications.

6 The title Neuroculture Collective speaks to autistic activism and political shifts within the autism community to paradigms of neurodiversity, and specifically references Francisco. Ortega (Editor) and Fernando Vidal (Editor), Neurocultures: Glimpses into an Expanding Universe (2011).

7 In the executive summary the authors write that ‘Collective Wisdom is “a hybrid field study that sets out to map, define, and shed light on co-creation methods within media (arts, documentary, and journalism) and adjacent areas of knowledge (design, open-source tech, urban and community planning).
